# Why a Condensate Can Be Thought of as Having a Definite Phase

**DOI:** 10.6028/jres.101.059

**Published:** 1996

**Authors:** S. M. Barnett, K. Burnett, J. A. Vaccaro

**Affiliations:** Department of Physics and Applied Physics, University of Strathclyde, Glasgow, G4 0NG, UK; Department of Physics, Clarendon Laboratory, Parks Road, Oxford, OX1 3PU, UK; National Institute of Standards and Technology, Gaithersburg, MD 20899-0001 USA; Physics Department, The Open University, Milton Keynes, MK7 6AA, UK

**Keywords:** Bose-Einstein condensate, broken symmetry, coherent state, open quantum system, phase

## Abstract

We present an argument for assigning a definite phase to an assembly of Bose-Einstein Condensed atoms. This relies on the demonstration that a coherent state of the condensed system is a robust state in the presence of interactions between the condensate and its environment.

## 1. Introduction

In this paper we shall discuss our view of why an assembly of Bose-Einstein condensed atoms may be considered to have a definite phase. This seems to be a long-standing problem as well as a rather general one; the analogous situation seems to arise in a wide range of systems exhibiting spontaneously broken symmetry, including the laser, superconductors, superfluids as well as particle physics [1]. Quite a body of work exists on the problem of the phase of a condensate and some (albeit a rather small part) of it will be discussed below. We present what we contend is a rather straightforward but hopefully convincing analysis of the problem. However, while parts of the argument seem to exist in the literature, we have not previously found our idea expressed in a direct form for the problem at hand. Our simple idea can be summed up in one sentence. *A Bose-Einstein condensate has a preferred phase because it is meaningful, on a macroscopic timescale, to ascribe one to it.* Clarifying and justifying this statement requires no new mathematics or physical theory.

This paper is laid out as follows. In Sec. 2 we give a brief account of what we see as critical comments in previous work. In Sec. 3 we discuss the nature of a condensate and why one would wish to assign it a phase. We then turn to the critical issue of robustness of description based on a pure state picture and show how that bears on the issue of assigning a phase. After that we examine the issue of survival of coherences and draw some conclusions.

## 2. Comments From Some Critical Earlier Works

We shall not be able to review the voluminous literature on this subject, but do want to draw attention to several issues raised by others that we believe are very much worth discussing further. We would first point to the work of Leggett and Sols [2]. These authors argue for a macroscopic wavefunction which has “an absolute phase” with the important qualification that “it is immediately and explicitly clear that this phase has no physical significance.” They also address the question of relative phases “for any given physical system the relative phase *θ* becomes ill-defined in the the limit *t* → ∞, but that the time (call it *τ_θ_*) for this to occur may well be long enough to allow, in principle at least, interesting experiments.” In our discussion we shall show in essence how *τ_θ_* can be defined and, what is more, how it can be related to the interaction between the condensate and its environment. They also pose, but do not resolve, the problem of “whether a measurement can *create* a relative phase when none previously existed ….” In summary they state “… the absolute phase of a superfluid is not a necessary, or indeed meaningful concept. However, under certain conditions, the relative phase of two superfluids can be meaningful, even when they are physically separated; but these conditions are extremely stringent.” We believe we shall present a “canonical” resolution of these questions.

Perhaps we should emphasise that so called “Condensed” systems come in many varieties and arise from a number of distinct broken symmetries. Peierls, for example [3], describes no fewer than three different distinct types. In one case at least, that of the laser, it is clear that the relative phases of the two lasers is meaningful and interference can be clearly demonstrated. Our contention is that the same should be true for condensates. In particular, we would argue that in the case of Bose-condensed assemblies of atoms the broken symmetry shares this critical feature with the laser.

The other discussion we would like to point to is that by Javanainen [4]. It is based on a proposed relation between quantum statistics and classical stochastic processes. Javanainen expresses the following interpretative hypothesis: “In any individual experiment the quantum system executes one realisation of the classical stochastic process.” Following this reasoning we would say that a harmonic oscillator in a highly excited number state “traverses a classical trajectory, with an initial phase that varies randomly from realisation to realisation.” This is again close to but not strictly equivalent to the point we want to make. Javanainen’s analysis leads us further to say that for the case of two separate condensates, a relative phase between the condensates exists (even if they both have a precisely defined number of particles) because the analogous classical stochastic process ascribes to each condensate a fixed (but random) phase. Hence two condensates would have a fixed (but random) phase difference. Javanainen also comments on the use of a commonly known trick [5, 6], i.e., idea of a symmetry-breaking field with a Hamiltonian of the form

$$H=\lambda {\hat{a}}^{+}+\lambda \;*\;\hat{a}$$ (1)

where *λ* = |*λ*|e^−^*^i^*^θ^ and $\hat{a}$ is the boson annihilation operator.

This term is often used in order to avoid the fact that for an isolated system no particular phase is chosen. The introduction of a term of this sort into the Hamiltonian for a system of bosons leads to a nonzero value $<\hat{a}>$ which remains for *N* → ∞, *then λ* → 0. This method works, as Gunton and Buckingham have shown [6], because the response to such a perturbation diverges as the transition point is approached in the thermodynamic limit. For finite systems, however, we have to ask how such an interaction would come about. Javanainen notes however that “the symmetry breaking field is purely fictitious: no physical interaction is known that leads to the Hamiltonian (above) ….” Such a term was also introduced [7] in the study of the analogy between the theory of a laser and a phase transition. In this case one can, in fact, think about this interaction as being due to an externally injected classical field. This again sidesteps the critical issue of how such a classically definable object comes about! Javanainen also briefly suggests a coherent histories approach and discusses the role of measurement and pointer bases as a link between quantum statistics and a classical stochastic process. However, his approach does not invoke measurement but “Instead, I suggest that if the correlation functions of a position agree with the correlation functions of a classical stochastic process, then let us say that position *is* a classical stochastic process.” For Javanainen it is in effect quantum jumps which “neatly yield spontaneously broken symmetries.” This approach of Javanainen goes some way to a satisfactory explanation but not quite far enough in our opinion. Two questions clearly arise from this approach: Firstly, what if the statistics don’t agree with those for a classical stochastic process? A number of approaches have in fact been made to cast quantum mechanics in this form [8, 9]. The second and perhaps most substantial problems is that the state accessed by a quantum jump is dependent on how we model the effect. For example, the different approaches to treating open systems, using stochastic wavefunction simulations, tend to localise the system on different types of pure state [10,11,12].

## 3. The Nature of a Condensate

Before addressing the origin of the condensate phase, we should establish more clearly what we mean by a condensed system. We shall take the following simple view as our starting point: A condensate occurs (or perhaps, can occur) when a large (macroscopic) number of bosons are put into the same one particle (first quantized) state. This is reasonable as a starting point, at least for trapped atoms and laser fields (in the laser the “first quantized state” is simply the cavity mode). The following three points concerning the nature of a condensate are almost obvious. However our discussion of condensate phase which follows relies on them, so we believe it is important to state them clearly:

1. A condensate is a quantum system. It follows, therefore, that its properties depend in large part on the measurements you can perform on it.
2. Condensate are *open systems*; some dynamical processes cause members of the condensate to be lost while other processes add particles to it.
3. It follows from (2) that condensates are not made in pure states. At the most fundamental level, there should be a highly entangled state of the particles forming the condensate plus the environment. It inevitably follows that the best way we can find by way of complete *a priori* description is a mixed state described by a density matrix. Typically, if not always, this will be diagonal in the particle number representation. It follows that we can predict (perhaps quite accurately) the particle number statistics *but* all we can say about off-diagonal matrix elements is that their average vanishes.

The last comment (3) contains the real heart of the problem. We can interpret such a mixed state as a statistical mixture of pure states, that is as a probability that the system is in one of a number of pure states. For example, a thermally occupied harmonic oscillator or boson mode has the density matrix

$${\hat{\rho }}_{\text{thermal}}=\sum_{n=0}^{\infty }\frac{{\bar{n}}^{n}}{{\left(\bar{n}+1\right)}^{n+1}}|n\rangle \;\langle n|.$$ (2)

Where |*n*〉 *is a number state and* $\bar{n}$ is the mean number. One can interpret this in the following way [13]. The oscillator is in a number state. The probability that this number state is |*n*> is then simply $\frac{{\bar{n}}^{n}}{{\left(1+\bar{n}\right)}^{n+1}}$. This interpretation is certainly bourne out if we measure the excitation number for each member of a large ensemble. It is, however, important to realise there are other interpretations where we consider each member of the ensemble to be in one of a different set of pure states. For example, we can also write the state of our system in the form

$${\hat{\rho }}_{\text{thermal}}=\frac{1}{\pi \bar{n}}\int d^{2}\alpha \exp\left\{-\frac{{\left|a\right|}^{2}}{\bar{n}}\right\}|a\rangle \;\langle a|$$ (3)

where |*a*〉 is the coherent state and the integration runs over the whole complex plane. We can then interpret $\frac{1}{\pi \bar{n}}\exp\left\{-\frac{{\left|a\right|}^{2}}{\bar{n}}\right\}$ as the probability density for a given coherent state to be occupied. Actually we should be careful as the coherent states are not orthogonal and so treating this as a probability is risky. We can, however, treat the quantity

$$\begin{array}{c} \frac{1}{\pi }\langle a\left|{\hat{\rho }}_{\text{thermal}}\right|a\rangle =Q_{\text{thermal}}\left(a\right)\\ =\frac{1}{\pi \left(\bar{n}+1\right)}\exp\left\{\frac{-{\left|a\right|}^{2}}{\bar{n}+1}\right\} \end{array}$$ (4)

as a probability density. Subtleties like this are not really the point; the point is what is the best state representation: and this is the question we shall answer. We will find that the coupling to the environment leads us to identify the coherent state as the best representation.

To sum up: the best *a priori* description of a condensate (or a laser mode) is a mixed state with a density matrix that is diagonal in the particle number representation. This can be interpreted as the probability for finding the system in a given pure state *but* in any of an infinite number of different bases. How is it then that the fields of each condensate (laser mode) seem to “have” a preferred (albeit random) phase leading to a nonzero expectation value for the field? This, in essence, is the condensate phase problem. Our solution to it involves the interaction with the environment and the concept of “robustness.”

## 4. Robustness and the Role of the Environment

We have noted that any *a priori* description of the condensate is at best a mixed state. The question we now address is “Is there a good pure state description?” To answer this we need a criterion for “best.” Our criterion for “best” is how long the state will survive in the open environment of the condensate. This is closely related to the question of predictability for an open quantum system [14, 15]. A number state description with the condensate in a eigenstate of particle number will only survive until one particle is lost or added; at this point it is in a state orthogonal to the initial one. This is obviously a very fragile state when exposed to the environment. A coherent state, however, might be expected to live longer. Long-lived states are termed (by us) “robust states” (see also Ref. [15]) and form a more natural or better pure-state description than short-lived states. In this section we give a more precise measure of “robustness” and determine states that are likely to be robust. Our measure of robustness is based on the overlap between the initial state and the density matrix it evolves to under the influence of the environment. Robust states look like the initial state for an appreciable time, i.e., they are a faithful likeness of the initial state for this time. This likeness can be measured simply and termed fidelity *F.* We should note that it would also be possible to define it in terms of the overlap between the evolved state and the state it would have evolved into in the absence of coupling to the environment. For the moment we will use the following simple overlap form given by Schumacher [16]

$$F=\langle \psi \left|\hat{\rho }\left(t\right)\right|\psi \rangle .$$ (5)

Here |*ψ*〉 is the initially chosen pure state and $\hat{\rho }\left(t\right)$ is the density operator it evolves into in the environment of the condensate. It is clear that from this expression that the fidelity, *F*, will always be less than or equal to unity, attaining its maximum value if and only if

$$\hat{\rho }\equiv |\psi \rangle \;\langle \psi |.$$

It is also clear that coupling to the environment will in general make $\hat{\rho }$ tend to a mixed state and cause *F* to evolve towards some nonzero value given by $\langle \psi \left|\hat{\rho }\left(\infty \right)\right|\psi \rangle$ where $\hat{\rho }\left(\infty \right)$ is the steady state density matrix. The speed at which *F* decays is what we shall take our measure of robustness. As long as *F* is significant, |*ψ*〉 will remain a reasonable description of the state. Robustness should be considered as a property of the evolution of *F*. However, for the sake of comparison it is useful to have simple numerical quantities. We therefore introduce two such quantities first the fidelity loss rate *L* given by

$$L=-\frac{dF}{dt}|t=0.$$ (6)

This is quite simply related to Gallis’ linear-entropy production rate [15] and is straightforward to calculate but is only meaningful for short times. A second quantity, more difficult to calculate but perhaps more meaningful, is the fidelity time defined by

$$F\left(\tau_{\text{Fid}}\right)=e^{-1}.$$ (7)

In order to see how these ideas work, we start with something very simple—the damped Boson-mode for which the evolution of the density operator is given by

$$\dot{\hat{\rho }}=\Gamma \left(2\hat{a}\hat{\rho }{\hat{a}}^{+}-{\hat{a}}^{+}\hat{a}\hat{\rho }-\hat{\rho }{\hat{a}}^{+}\hat{a}\right).$$ (8)

The fidelity is again given by

$$F=\langle \psi \left|\hat{\rho }\left(t\right)\right|\psi \rangle$$ (9)

and so the fidelity loss rate is

$$\begin{array}{c} L={-\langle \psi \left|\dot{\hat{\rho }}\right|\psi \rangle |}_{t=0}\\ =-\Gamma \left\{2\langle \psi \left|\hat{a}\right|\psi \rangle \;\langle \psi \left|{\hat{a}}^{+}\right|\psi \rangle -2\langle \psi \left|{\hat{a}}^{+}\hat{a}\right|\psi \rangle \right\}\\ =2\Gamma \left\{\langle \psi \left|{\hat{a}}^{+}\hat{a}\right|\psi \rangle -{\left|\langle \psi \left|\hat{a}\right|\psi \rangle \right|}^{2}\right\}. \end{array}$$ (10)

This is clearly positive semi-definite since

$$\begin{array}{c} \langle \psi \left|{\hat{a}}^{+}\hat{a}\right|\psi \rangle -{\left|\langle \psi \left|\hat{a}\right|\psi \rangle \right|}^{2}\\ =\langle \psi |\left\{{\hat{a}}^{+}-\langle \psi \left|{\hat{a}}^{+}\right|\psi \rangle \right\}\left\{\hat{a}-\langle \psi \left|\hat{a}\right|\psi \rangle \right\}|\psi \rangle \end{array}$$ (11)

which is the squared modulus of the state

$$\left\{\hat{a}-\langle \psi \left|\hat{a}\right|\psi \rangle \right\}|\psi \rangle .$$

The fidelity loss rate is thus *minimised* for states with

$$\hat{a}/\psi \rangle =\langle \psi \left|\hat{a}\right|\psi \rangle |\psi \rangle ,$$ (12)

i.e, right-eigenstates of $\hat{a}$. These states are, of course, the coherent states. This implies that

L{|a〉}=0 (13)

and hence that the coherent states are, at least for short times, robust against decay (see Refs. [14] and [15].

The states with the most rapid loss of fidelity will be those with $\langle \psi \left|\hat{a}\right|\psi \rangle =0$ (such as the number states) for which

L{|n〉}=2Γn. (14)

It seems that maximising <*a*> minimises *L*. As $\hat{a}|a\rangle =\hat{a}|a\rangle$ it is clear that the coherent states *alone* minimise *L*. This suggests that they are excellent candidates for the best pure state description. It would be wrong, of course, to assume that *L* = 0 means *F* always remains large. We can and should look to longer ties to check on the decay of *F* and hence find the fidelity time. This is what we proceed to do now.

The master equation [Eq. (8)] can be solved exactly for any chosen initial state. For an initial coherent state |*a*〉 or number state |*n*〉 the density matrices are

$$\begin{array}{c} \hat{\rho }\left(0\right)=|a\rangle \;\langle a|,\\ \to \hat{\rho }\left(t\right)=|ae^{-\Gamma t}\rangle \;\langle ae^{-\Gamma t}| \end{array}$$ (15)

$$\begin{array}{c} \hat{\rho }\left(0\right)=|n\rangle \;\langle n|\to \hat{\rho }\left(t\right)=\\ \sum_{l=0}^{n}{\left(e^{-2\Gamma t}\right)}^{n-l}{\left(1-e^{-2\Gamma t}\right)}^{l}\frac{n!}{l!\left(n-l\right)!}|n-l\rangle \;\langle n-l|. \end{array}$$ (16)

The corresponding fidelities follow directly from the overlap between two coherent states [17] and the orthonormality of the number states. We find

$$F\left(|\psi \rangle \right)={\left|\langle \psi |ae^{-\Gamma t}\rangle \right|}^{2}=\exp\left\{-{\left|a\right|}^{2}{\left(1-e^{-\Gamma t}\right)}^{2}\right\}$$ (17)

and

$$F\left(|n\rangle \right)=\langle n\left|\hat{\rho }\left(t\right)\right|n\rangle =e^{-2\Gamma nt}.$$ (18)

We are primarily concerned with large particle numbers which implies small *Γτ_Fid_*. This means we can set $1-e^{-\Gamma_{\text{Fid}}}≅\Gamma \tau_{\text{Fid}}$

$$F\left(|a\rangle \right)\sim \exp\left\{-{\left|a\right|}^{2}\Gamma^{2}t^{2}\right\}$$ (19)

for short times. The resulting fidelity times are then

$$\begin{array}{r} \tau_{\text{Fid}}\;\left(|n\rangle \right)=\frac{1}{2n\Gamma }\\ \tau_{\text{Fid}}\;\left(|a\rangle \right)\sim \frac{1}{\Gamma \left|a\right|}. \end{array}$$ (20)

Note that $\bar{n}={\left|a\right|}^{2}$, so that the fidelity time for a coherent state is $2{\bar{n}}^{1/2}$ times larger for a coherent state with about the same number of particles. The smallest condensates might have $\bar{n}\sim 10^{3}$ for which this factor is about 60. Actually, the situation for the mean field description is *better* even than this, for if we have decided that $\langle \hat{a}\rangle =a$, we find that this “mean field” decays still even more slowly as

$$\langle \hat{a}\left(t\right)\rangle =ae^{-\Gamma t}.$$

There is now a hierarchy of decay rates

$$\begin{array}{l} 2n\Gamma —\text{rate of changing number state}\\ {\bar{n}}^{1/2}\Gamma —\text{rate of leaving coherent state}\\ \Gamma —\text{rate of decay of mean field}. \end{array}$$ (21)

In more interesting systems, the dynamics will be complicated but for large occupation numbers we will consistently find the same story which can be summarised as follows. A useful pure state description needs to be long-lived and hence *robust*. Robustness requires a large value of $\langle \hat{a}\rangle$, and this means coherent states (or states very like them). Perhaps we should mention at this point that for the Scull-Lamb laser model [7] the nonlinear saturation tends to clamp the magnitude of the field so that it can only change by phase diffusion. The result is that (i) number states change quickly, (ii) coherent states for which |*a*|^2^ is very different to ${\bar{n}}_{\mathrm{ss}}$ change less quickly (iii) coherent states for which ${\left|a\right|}^{2}\sim {\bar{n}}_{\mathrm{ss}}$ are highly robust. This is reflected in the fact that (Ref. 7)

$$\langle \hat{a}\rangle \sim {\bar{n}}_{\mathrm{ss}}^{1/2}e^{i\theta }\exp\left[-\frac{1}{4}\cdot \frac{2\Gamma G}{{\bar{n}}_{\mathrm{ss}}^{1/2}}\cdot t\right].$$ (22)

Here *G* is the gain of the laser. Note this decays at a rate *slower* than for straight decay. Hence the excellent approximation of the laser mode by a coherent state.

Let us now consider briefly a more complicated coupling to the environment more suited, perhaps, to describing a trapped atom condensate. This can be written in the form

$$\begin{array}{c} \dot{\hat{\rho }}=-i\left[{\hat{H}}_{\mathrm{NL}},\hat{\rho }\right]\\ +\Gamma_{2}\;\left(2{\hat{a}}^{2}\hat{\rho }{\hat{a}}^{+2}-{\hat{a}}^{+2}{\hat{a}}^{2}\hat{\rho }-\hat{\rho }{\hat{a}}^{+2}{\hat{a}}^{2}\right)\\ +\Gamma_{1}\;\left(2\hat{a}\hat{\rho }{\hat{a}}^{+}-{\hat{a}}^{+}\hat{a}\hat{\rho }-\hat{\rho }{\hat{a}}^{+}\hat{a}\right)\\ +{\tilde{\Gamma }}_{1}\;\left(2+\hat{a}\hat{\rho }a-\hat{a}{\hat{a}}^{+}\hat{\rho }-\hat{\rho }\hat{a}{\hat{a}}^{+}\right) \end{array}$$ (23)

Here ${\hat{H}}_{\mathrm{NL}}$ represents the nonlinear deterministic coupling. The term proportional to *Γ*_1_ represents one body loss. The ${\tilde{\Gamma }}_{1}$ term represents one body feeding (gain) of the condensate. The term multiplied by *Γ*_2_ provides two body loss, e.g., due to dipolar collisions in a trap.

It is too complicated to find the fidelity time but we can at least find *L*. We find

$$\begin{array}{c} L\left(|\psi \rangle \right)=\left\{\langle \psi \left|\{H_{2}\right|\psi \rangle \;\langle \psi \left|]\right|\psi \rangle \right\}\\ +2\Gamma_{2}\left\{\langle \psi \left|{\hat{a}}^{+2}{\hat{a}}^{2}\right|\psi \rangle -{\left|\langle \psi \left|\hat{a}\right|\psi \rangle \right|}^{2}\right\}\\ +2\Gamma_{1}\left\{\langle \psi \left|\hat{a}\hat{a}\right|\psi \rangle -{\left|\langle \psi \left|\hat{a}\right|\psi \rangle \right|}^{2}\right\}\\ +2{\tilde{\Gamma }}_{1}\left\{\langle \psi \left|\left(\hat{a}+\hat{a}\right)\right|\psi \rangle -{\left|\langle \psi \left|\hat{a}\right|\psi \rangle \right|}^{2}\right\}. \end{array}$$ (24)

The Hamiltonian term is precisely zero. As before *L* is minimised for the coherent states for which

$$L\left(|a\rangle \right)=2{\tilde{\Gamma }}_{1}.$$ (25)

At the other extreme, for the number states we find

$$L\left(|n\rangle \right)=2{\tilde{\Gamma }}_{1}n\left(n-1\right)\;2{\tilde{\Gamma }}_{1}n+2{\tilde{\Gamma }}_{1}\left(n+1\right).$$ (26)

We note that this result shows the important fact that number states are even *more sensitive* to two body loss than to one body loss.

The analysis for times *t* > 0 is best carried out in the transformed picture where the direct effects of the deterministic coupling are eliminated. The required transformation is given by $|\psi \rangle \to \hat{U}|\psi \rangle$ where $\hat{U}=\exp\;\left(i{\hat{H}}_{NL}t\right)$. The detailed analysis in the transformed picture is beyond the scope of this paper although we note that when ${\hat{H}}_{NL}$ is a functions of ${\hat{a}}^{†}\hat{a}$ *t*he net result is, in general, enhanced phase diffusion. For these cases coherent states remain the robust states provided the coupling is not too strong.

## 5. Summary of Analysis

The master equation for the density matrix will tell us about the evolution and statistics for the condensate. the results we have obtained sow that it is meaningful to ascribe a mean field, $\langle \hat{a}\rangle$, associated with a pure state or nearly pure state to any given realisation of a condensate. This provides a justification for preferring a pure state (or near pure state) description in terms of states that are *robust*. A robust state will remain a reasonable description for a longer time than a nonrobust state. For bosons coupled to an environment, robust states are near to the coherent states (in general) in that they tend to have $\langle a+a\rangle \sim {\left|\langle \hat{a}\rangle \right|}^{2}$, and $\langle {\hat{a}}^{+2}{\hat{a}}^{2}\rangle \sim {\left|\langle \hat{a}\rangle \right|}^{4}$. The larger the system gets, the more important robustness will be; a number state lives only until one particle has been lost, but a coherent state lives much longer. The well-defined “phase” for a condensate thus follows from the fact that we can think of it as being in a state that is near to a coherent one. For a coherent state we find [18]

$$\begin{array}{r} \frac{\Delta N}{N}={\left(\bar{n}\right)}^{-1/2}\\ \Delta \phi \sim \frac{1}{2}{\left(\bar{n}\right)}^{-1/2}. \end{array}$$ (27)

These become increasingly well defined as $\bar{n}\to \infty$. The rapid decay of off-diagonal coherences in the coherent state basis is, of course, well-known [19]. The point being made in this paper is that it is also the reason why we can often ascribe a mean field to an open system! A condensate has a preferred phase, or a mean field because it is meaningful, on a macroscopic timescale, to ascribe one to it. Robustness also explains why using Bogoliubov’s trick works. As *n* → ∞ and λ → ∞, only a robust state will survive (i.e., a near coherent state). Furthermore this interaction generates coherent states. Other interactions would not push the system into coherent states and would therefore not lead to robust states.

## 6. Survival of Quantum Coherence

A condensate is an open quantum system. We can ask, however, if our pure state description is at all meaningful in light of the nonlinear mechanisms which can induce a superposition of coherent states. We are thinking in particular of the proposal of Wright, Walls and Garrison [20] that a condensate might exhibit quantum collapses and revivals of the mean field due to the anharmonic nature of the interaction between the atoms forming the *condensate has a preferred phase because it is meaningful, on a macroscopic timescale, to ascribe one to it.* same time-scale on which a number state decays. It seems that the very fact that we are working in a regime in which it is preferable to consider $\langle \hat{a}\rangle \neq 0$ and a coherent state description would tend to rule out the development of such short-lived quantum coherences. It is no doubt true that the ensemble average $\langle \hat{a}\rangle$ will decay to zero, or that given an initial $\langle \hat{a}\rangle$ we can only say that the future evolution will on average make it tend to zero. However, there are two processes which compete to do this; one is the quantum collapse, but the other is the coupling to the environment. The combination tends to diffuse the phase of a coherent state over a 2π interval in the collapse time π/(|*α*|*µ*). Here *µ* is a parameter that determines the strength of the nonlinearity and whose value reduces with increasing atom number. Thus for sufficiently large samples the collapse time is much longer than the fidelity time of a number state. The robust description of the condensate, therefore, is that of a coherent-like state undergoing phase diffusion.

## 7. Conclusions

This paper sets out a research programme for examining the issue of symmetry breaking in mesoscopic systems. Our conclusion in terms of a robust pure-state description based on coherent states may appear profound to some and obvious to others: it may or may not lead to experimentally testable conclusions. We believe in fact that it should be useful in assessing such important (and seemingly intractable) ideas as quantum measurements and the classical limit.

One further issue seems worth raising. The signature for a robust pure state description should also occur in wave-function Monte Carlo simulations. For a large highly excited system we should find that a method which tends to localise on coherent states [11] should lead to a steadier, less violently jumping evolution than an approach which tends to localise the energy [10]. Evidence supporting this point of view can be found in simulations of superposition’s of coherent states [12].

Quantum theory suggests that a complete description of our open system consists of a highly entangled wave-function with an enormous number of degrees of freedom. Such a wavefunction is impossible to calculate and, even if we knew its form, would be too unwieldy to be useful. A useful description must be based on the system of interest—in this case the condensate plus an environment. However, models based on this idea almost inevitably lead to a mixed-state description of the system of interest. Within this description we can ask it it is reasonable to interpret the mixed state as a probability for a given realisation of the system to be in a pure state. This is where the idea of robustness comes in. It is our contention that one can indeed interpret each system as being in a pure state *but* that this description is only useful it it changes slowly on a macroscopic time-scale.

In reality we should consider Schrödinger’s cat as being in a highly entangled superposition of nucleus undecayed plus a live cat and of nucleus decayed plus a dead one or, for the cat alone, statistical mixture of alive and dead. If we ask for a definitive statement about the state of the cat then we find it either alive or dead. That we do not (cannot) find it as either | *alive* 〉 + | *dead* 〉 *or* | *alive* 〉 − |*dead* 〉 is partly because we do not look for this possibility, but more so because such a state decays so rapidly due to the coupling to the environment. From our point of view Schrödinger’s cat is | *alive* 〉 *or* | *dead* 〉 because (as with condensate phase) it is meaningful, on a macroscopic timescale, to ascribe one of these properties to it!
